# Supplementary material for: The Carcinogen Cadmium Activates Lysine 63 (K63)-Linked Ubiquitin-Dependent Signaling and Inhibits Selective Autophagy
Source: Cancers (Basel). 2021 May 20;13(10):2490. doi: 10.3390/cancers13102490 (PMC8161291; doi:10.3390/cancers13102490)
Supplement: Supplementary file 1 [file cancers-13-02490-s001.zip › cancers-1211623-supplementary.pdf]

cancers

MDPI

The Carcinogen Cadmium Activates Lysine 63 (K63)-Linked Ubiquitin-Dependent Signaling and Inhibits Selective Autophagy

Abderrahman Chargui, Amine Belaid, Papa Diogop Ndiaye, Véronique Imbert, Michel Samson, Jean-Marie Guigonis, Michel Tauc, Jean-François Peyron, Philippe Poujeol, Patrick Brest, Paul Hofman, Baharia Mograbi

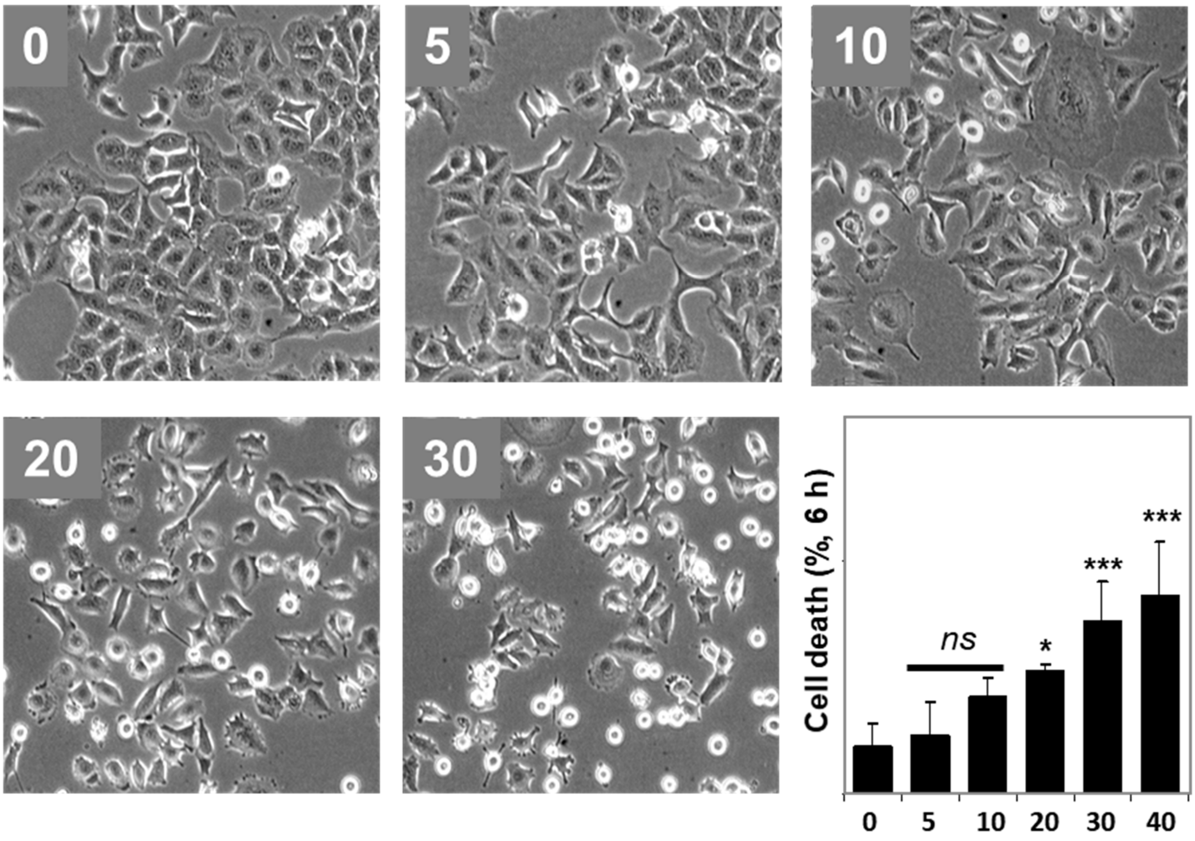

Figure S1. Dose-response viability of PCT cells after Cd treatment. PCT cells were stimulated with increasing Cd doses (μM); 6 h later, the cell morphology was then examined. Note that at 5 μM, Cd induced massive K63-linked ubiquitination, and meanwhile, it did not adversely affect cell viability. * $p < 0.01$, and *** $p < 0.0008$.

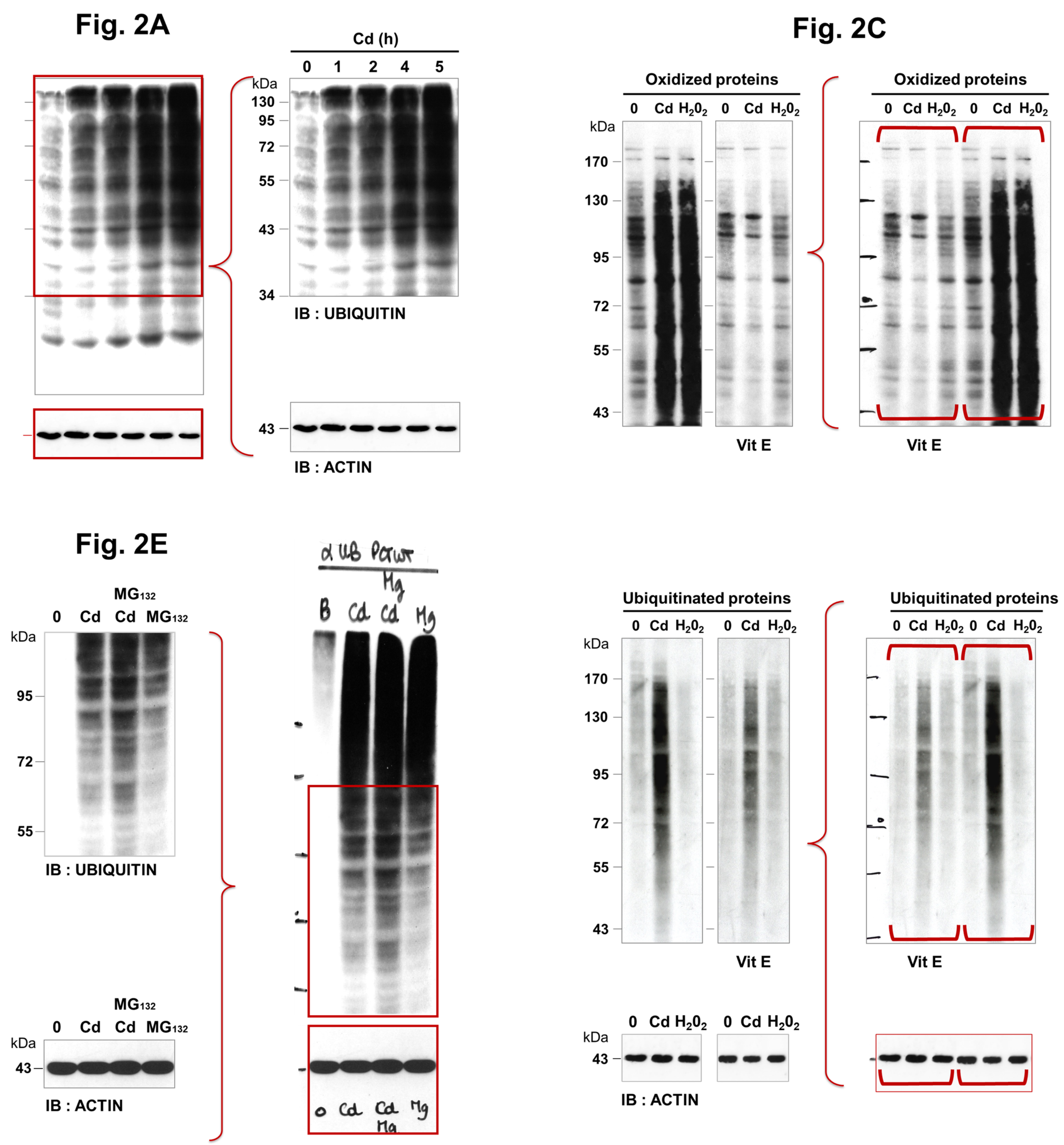

Figure S2. Uncropped images of immunoblots from Figure 2.

Fig. 3B

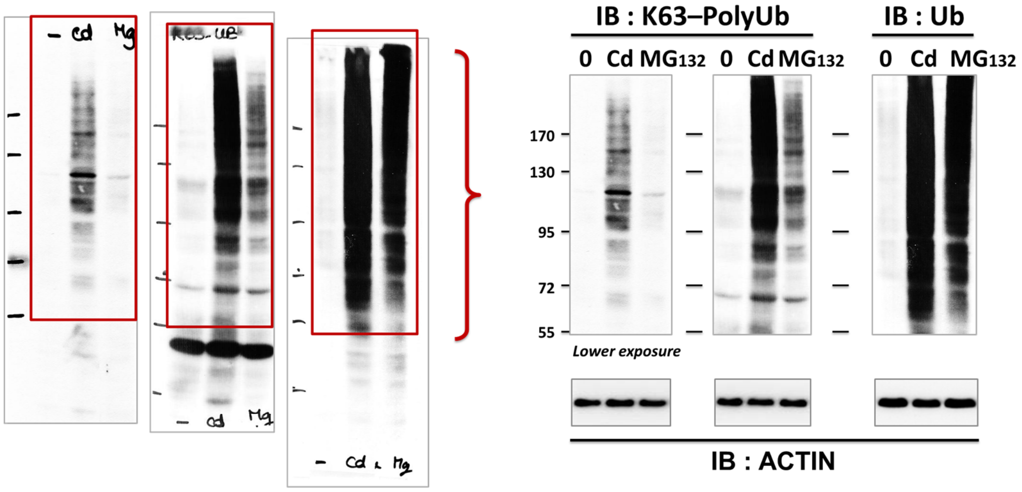

Fig. 3C

Fig. 3D

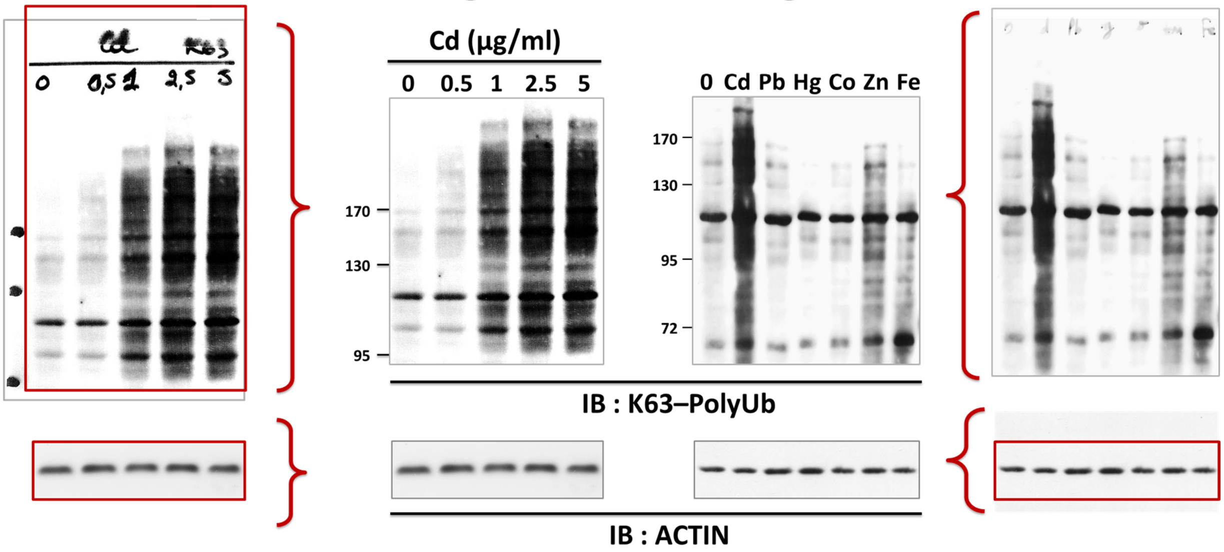

Fig. 3E

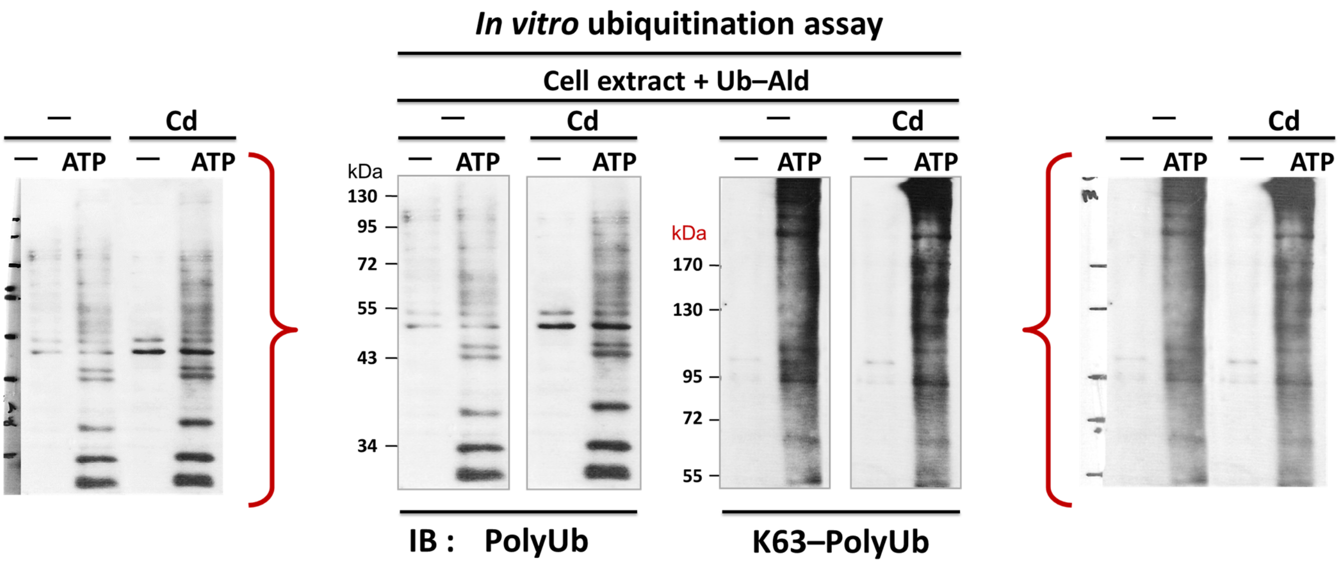

Fig. 3F

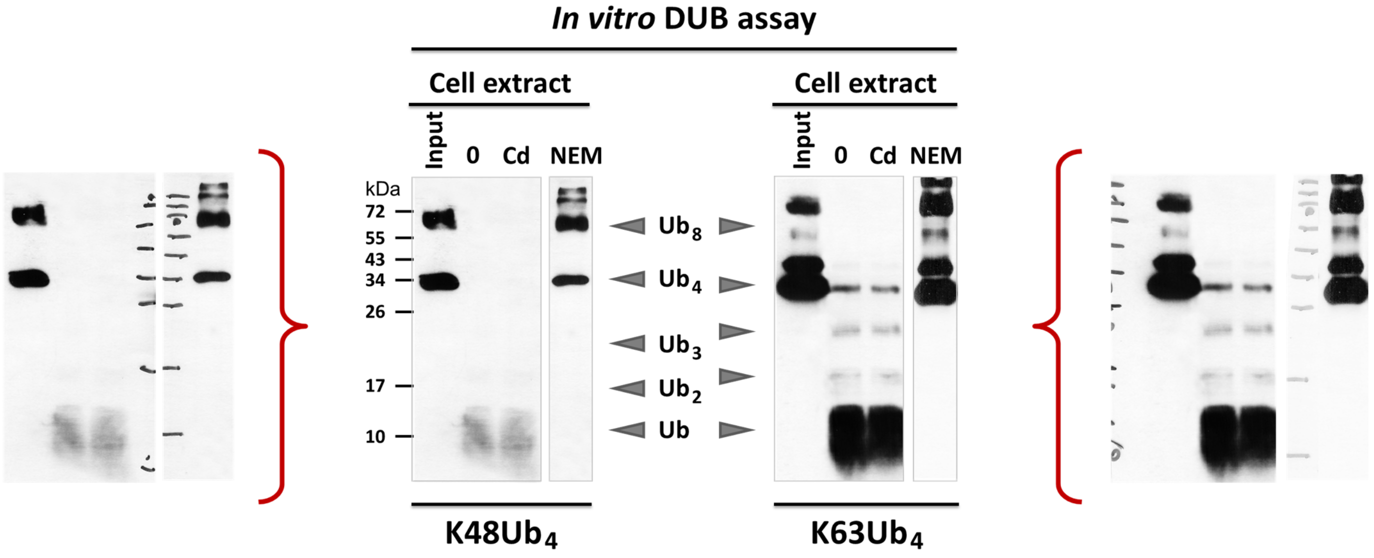

Figure S3. Related to Figure 3B–F. The red boxes indicate the cropped regions.

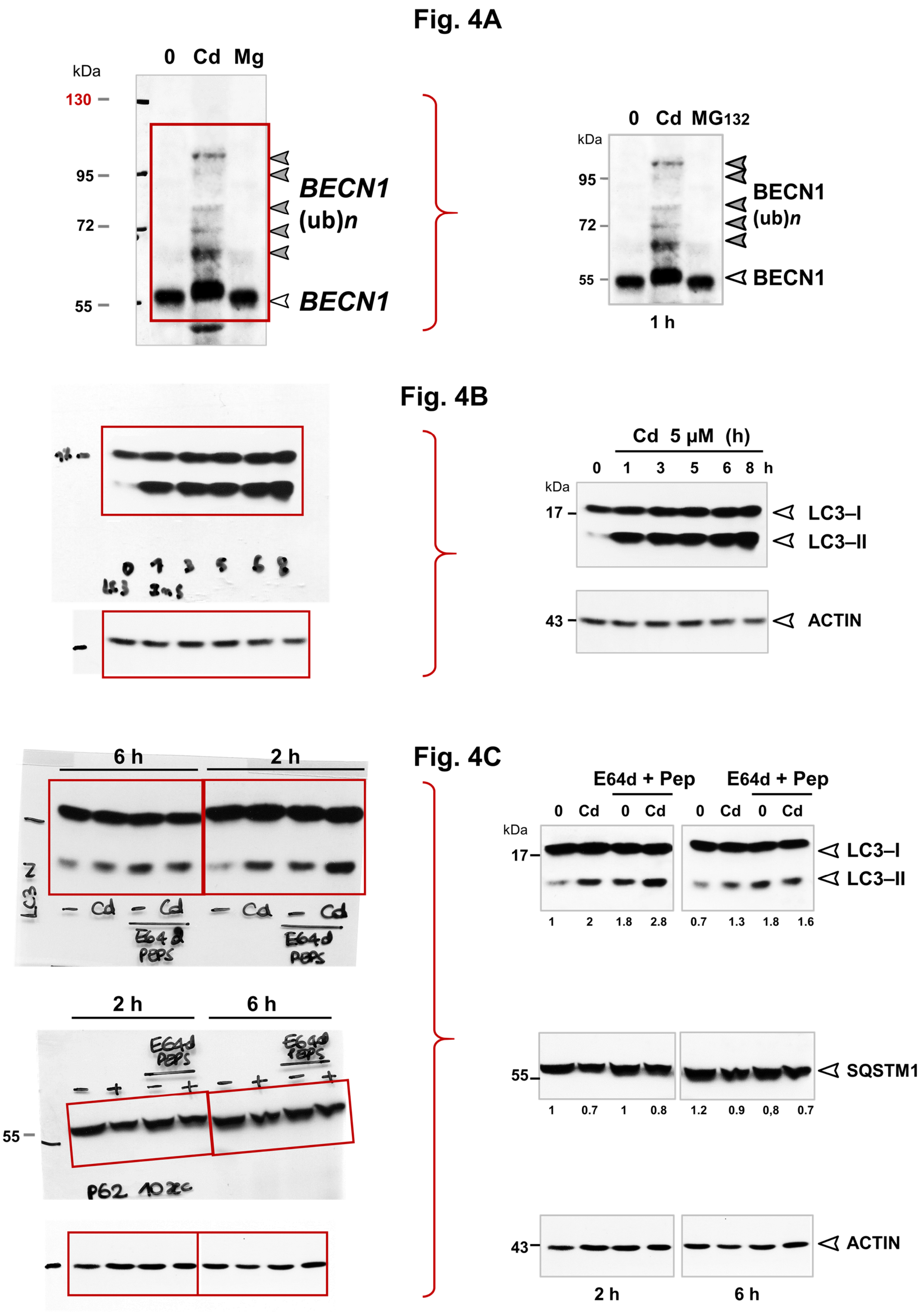

Figure S4. Related to Figure 4A–C. The red boxes indicate the cropped regions.

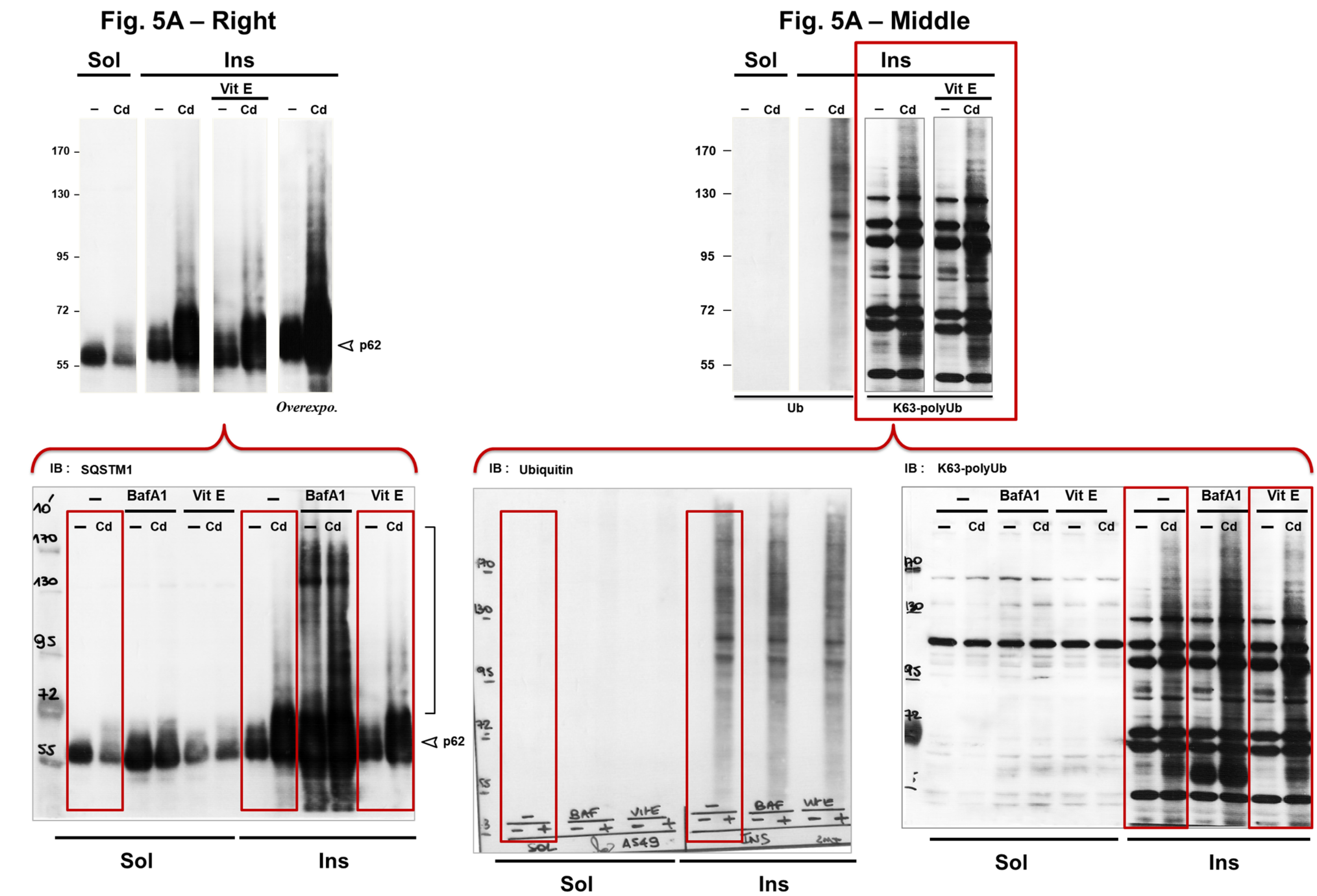

IB SQSTM1	Soluble	Insoluble
µg loaded	25 µg	25 µg
Exposure time	10 sec	10 sec

IB Ubiquitin	Soluble	Insoluble
µg loaded	75 µg	75 µg
Exposure time	2 min	2 min

IB K63-polyUb	Soluble	Insoluble
µg loaded	75 µg	75 µg
Exposure time	1.5 min	1.5 min

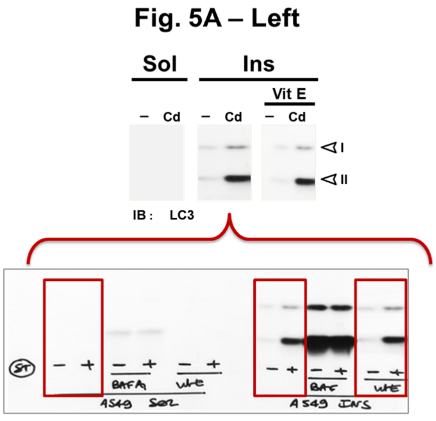

IB LC3	Soluble	Insoluble
µg loaded	50 µg	50 µg
Exposure time	30 sec	30 sec

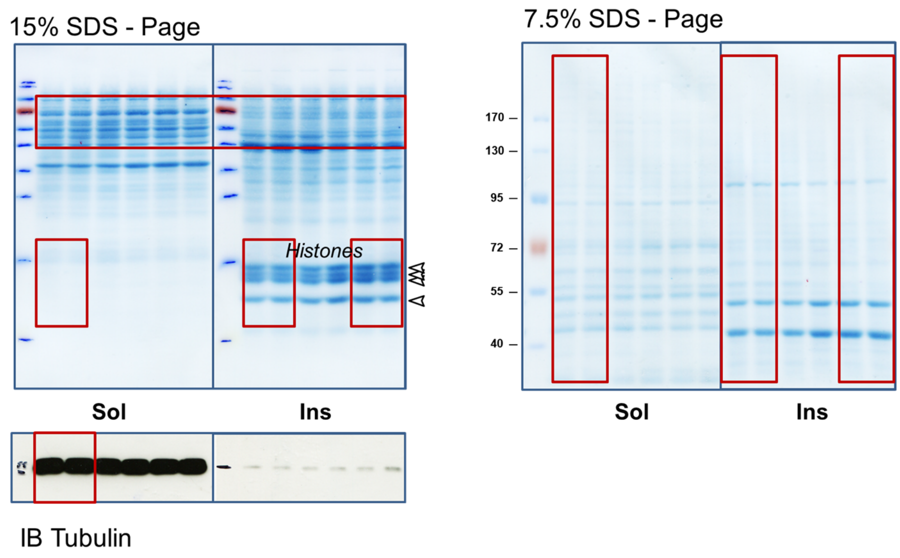

Figure S5. Related to Figure 5A. The red boxes indicate the cropped regions. Below, the amido black staining is used as a loading control and a control for contamination between the soluble and insoluble fractions (histone, arrowheads). Cross-contamination of insoluble and soluble fractions was also checked by immunoblotting with TUBULIN (cytosolic extracts). The amount of protein loaded onto the gel, and the exposure times of the films are indicated.

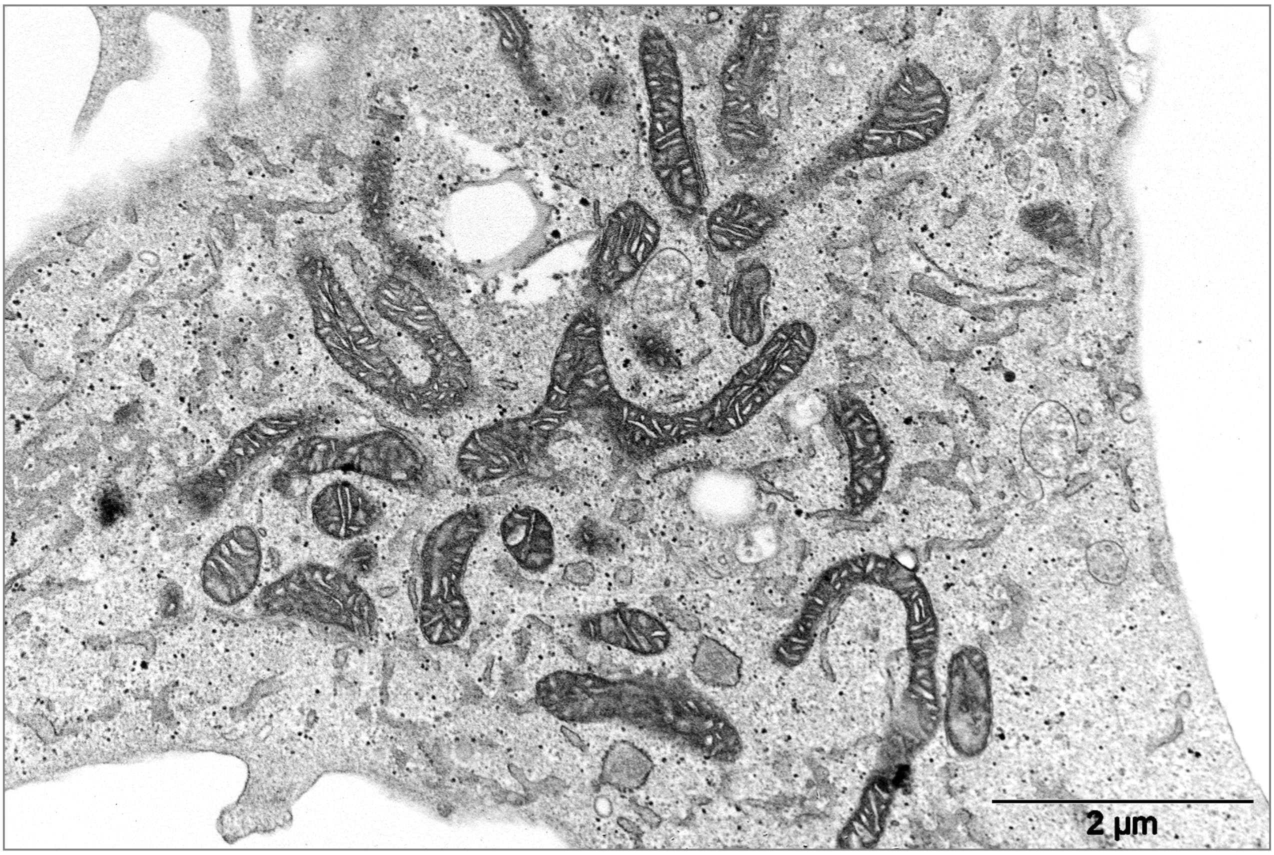

Figure S6. Related to **Figure 5** and **Figure 6**. Electron microscopic photographs of control cells**.**

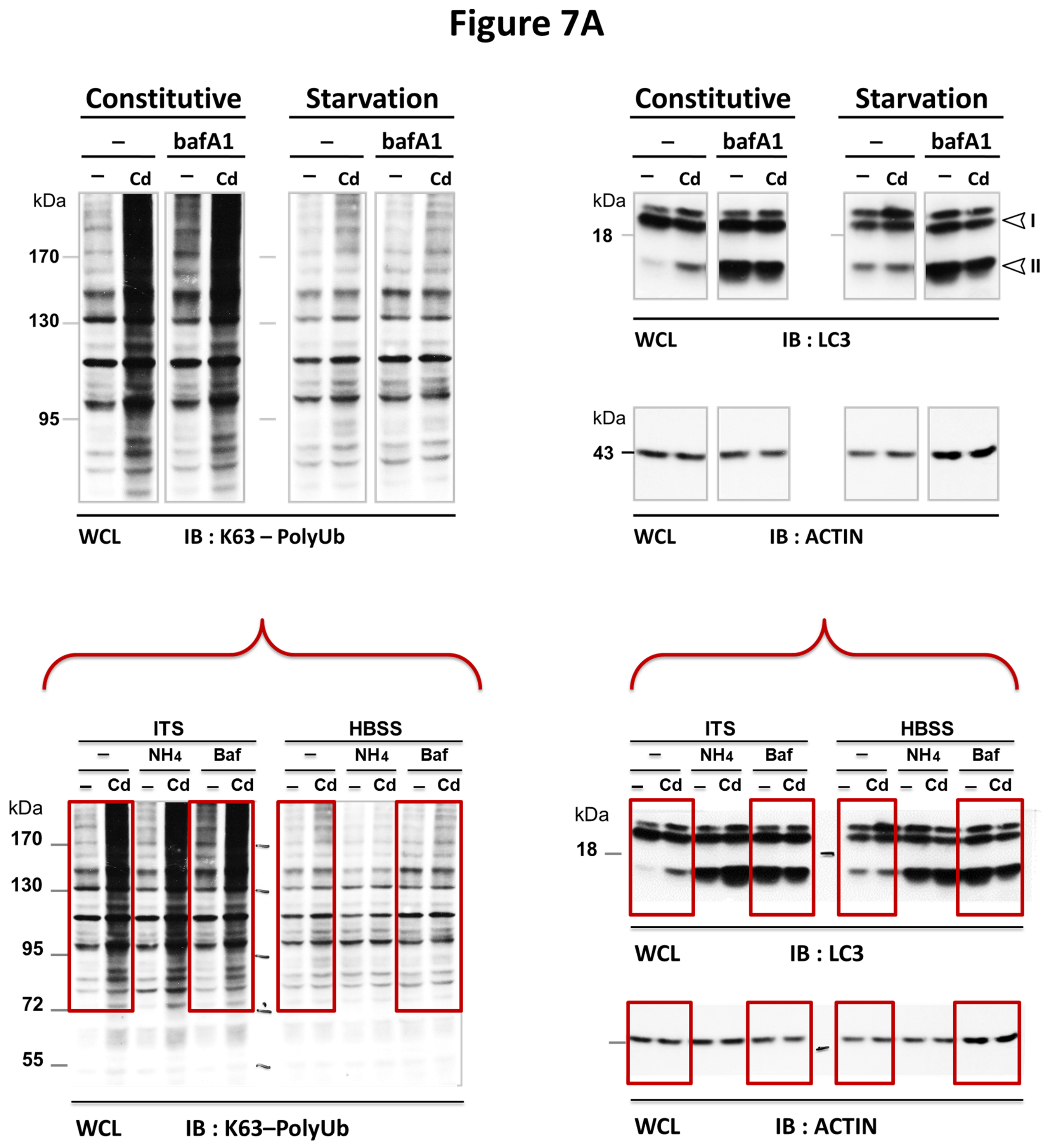

Figure S7. Full uncropped images of immunoblots from Figure 7A. The red boxes indicate the cropped regions.

Fig. 7B

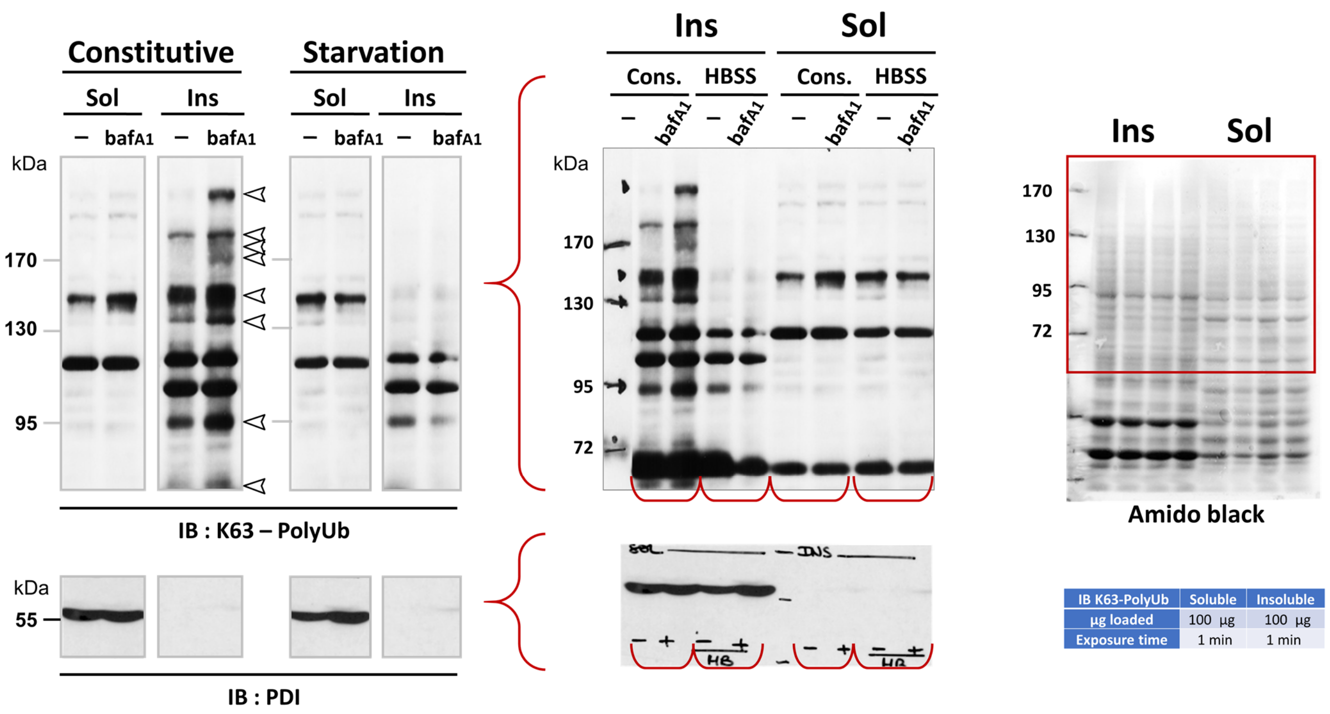

IB K63-PolyUb	Soluble	Insoluble
µg loaded	100 µg	100 µg
Exposure time	1 min	1 min

Fig. 7C

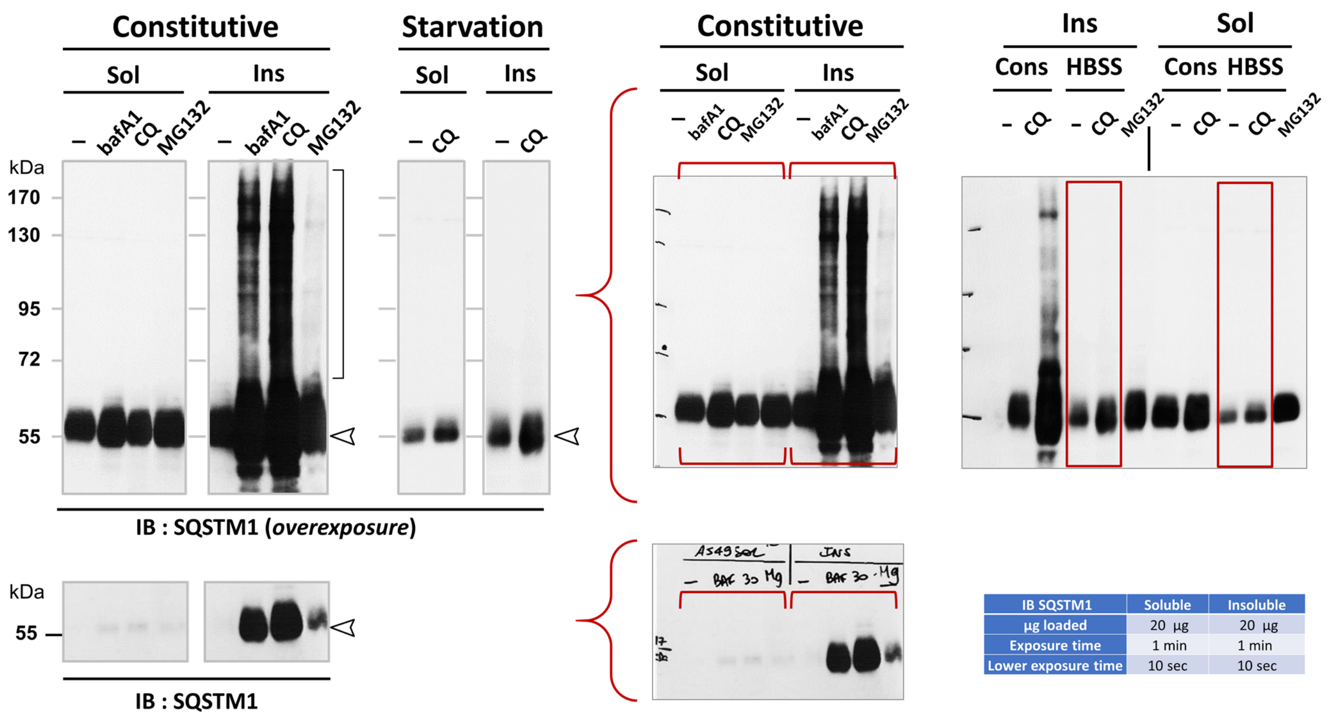

IB SQSTM1	Soluble	Insoluble
µg loaded	20 µg	20 µg
Exposure time	1 min	1 min
Lower exposure time	10 sec	10 sec

Figure S8. Full uncropped images of immunoblots from Figure 7B–C. The red boxes indicate the cropped regions. The amount of protein loaded onto the gels and the exposure times of the films are indicated.

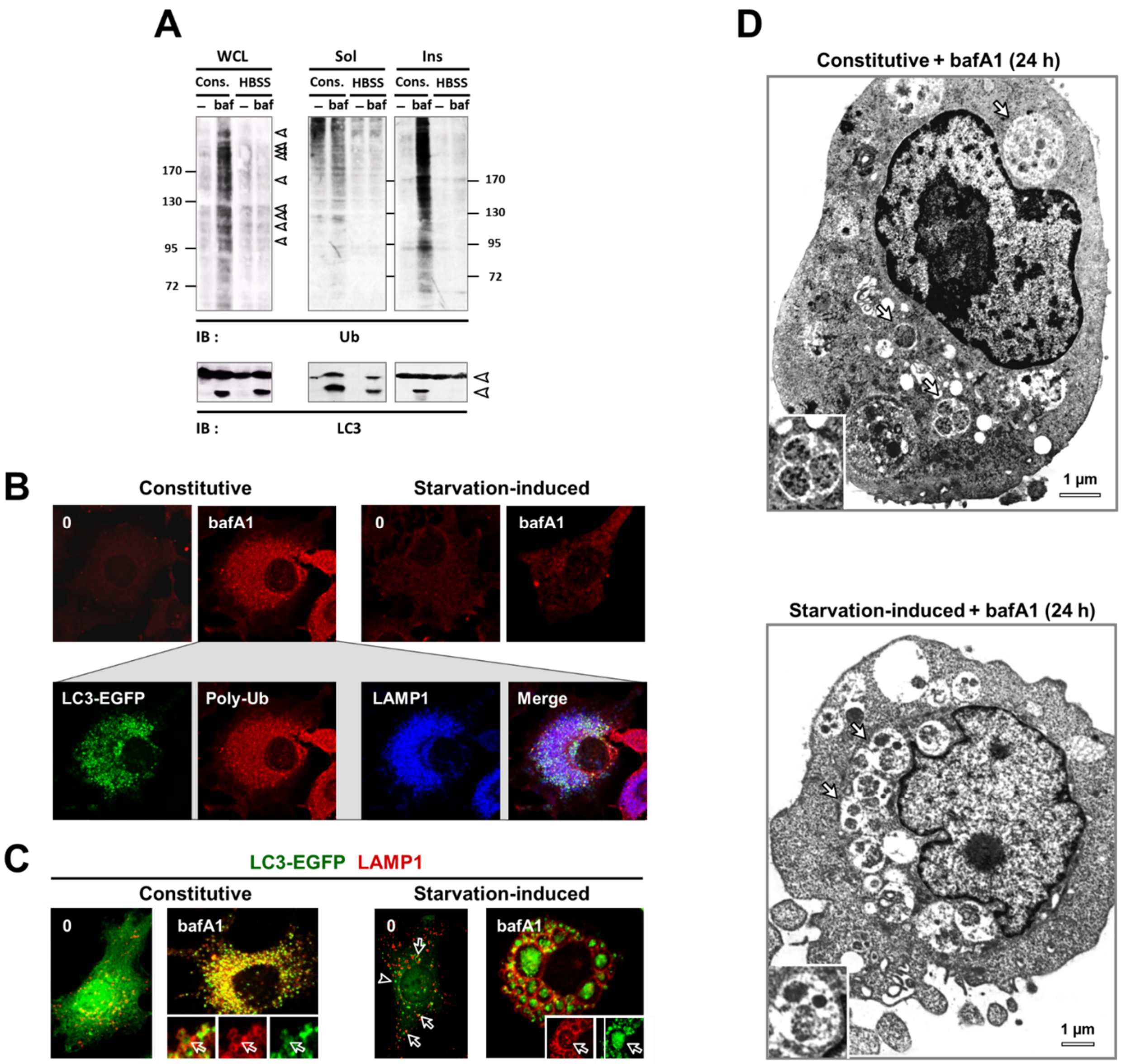

Figure S9. Degradation of ubiquitinated proteins by constitutive autophagy. To accumulate substrates within autolysosomal structures, autophagy was inhibited at the degradation step by bafilomycin A1 (bafA1, 100 nM) treatment of Sertoli cells, a cell type with a high level of autophagy flux. (**A**) Accumulation of ubiquitinated protein aggregates (insoluble pellet, 24 h). (**B**–**C**) Localization of ubiquitinated protein aggregates within autolysosomes (LC3-EGFP and LAMP1 positive; fluorescence images) in nutrient-rich conditions (constitutive or cons.). (**D**) Accumulation of single membrane autolysosomes with dense undigested bodies (arrows; inset; 24 h) under both conditions (electron microscopy micrographs). Note that the cargoes within the autolysosomes in starved and bafA1 conditions (HBSS + bafA1) were not tagged with ubiquitin (See Figure 6 and Figure S10).

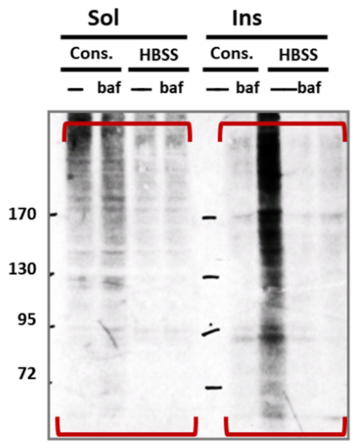

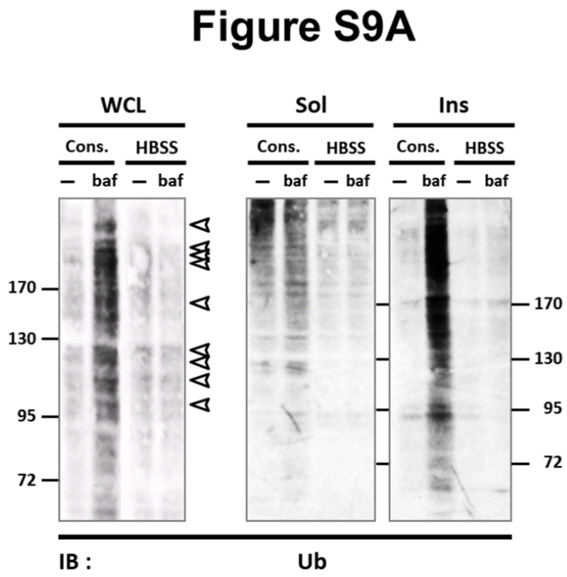

IB Ub	Soluble	Insoluble	WCL
μg loaded	100 μg	100 μg	100 μg
Exposure time	1 min	1 min	1 min

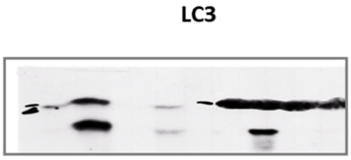

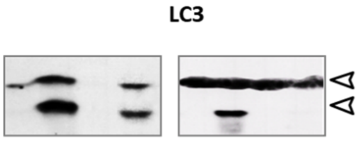

IB LC3	Soluble	Insoluble	WCL
μg loaded	50 μg	50 μg	50 μg
Exposure time	1 min	1 min	1 min

Figure S10. Full uncropped images of immunoblots from Figure S9A**.** The red boxes indicate the cropped regions. The amount of protein loaded onto the gels and the exposure times of the films are indicated.

Fig. 8A

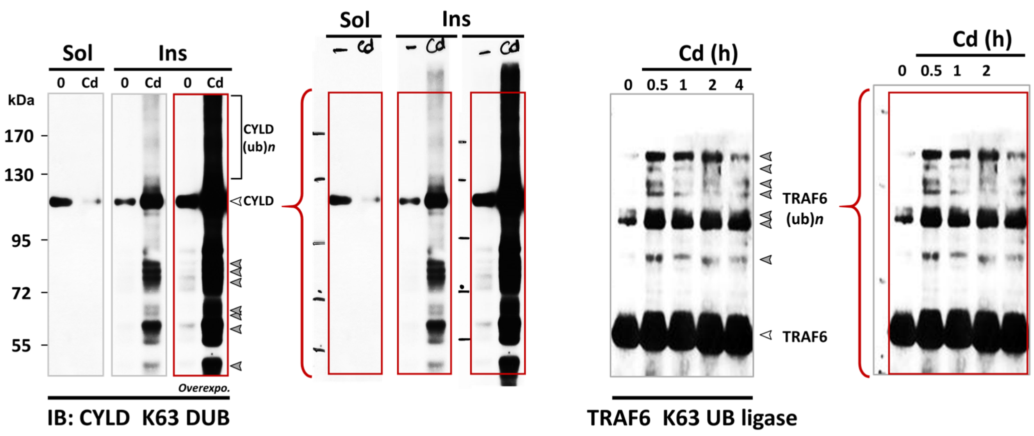

Fig. 8B

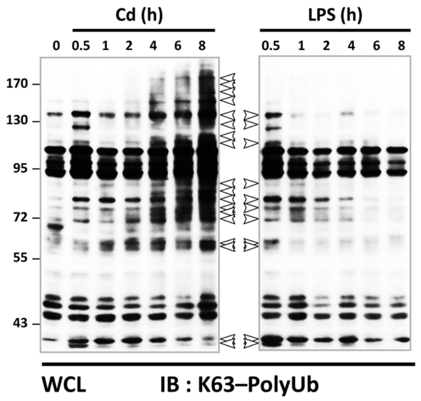

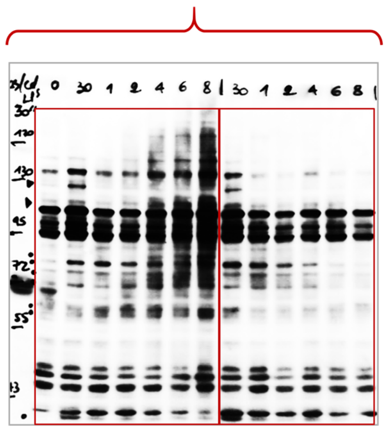

IB	K63–PolyUb
µg loaded	100 µg
Exposure time	2 min

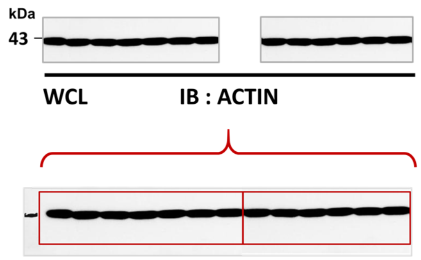

Fig. 8C

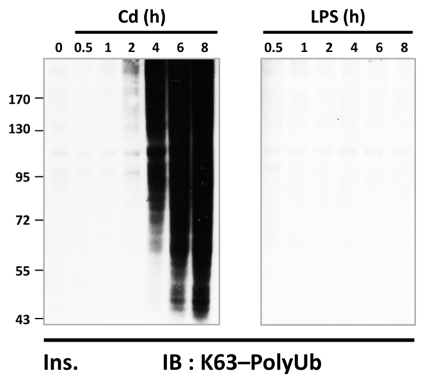

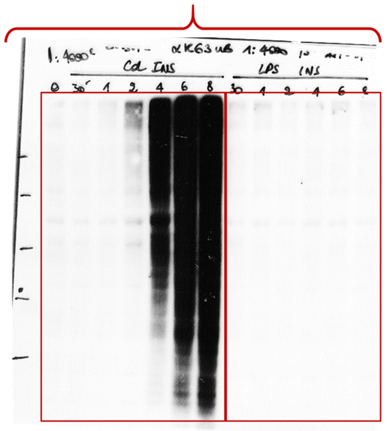

IB	K63–PolyUb
µl loaded	100 µg
Exposure time	1 min

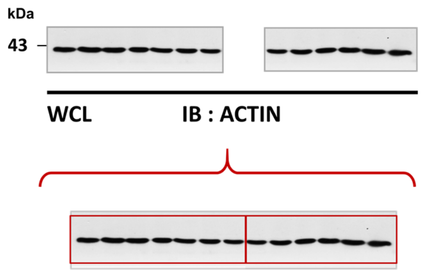

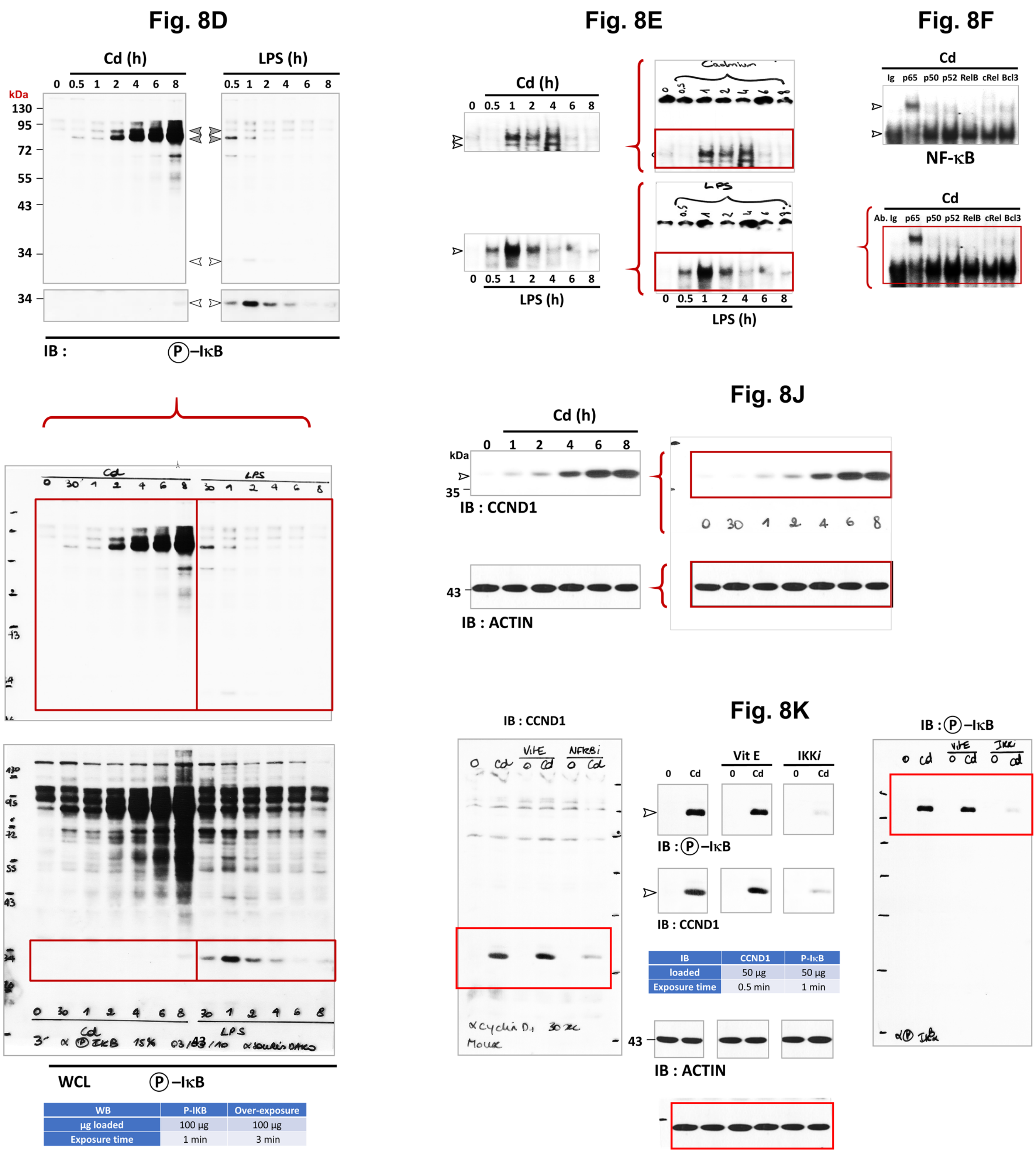

Figure S11. Related to Figure 8A–K. The red boxes indicate the cropped regions. The amount of protein loaded onto the gels and the exposure times of the films are indicated.
